# A Wild *C. Elegans* Strain Has Enhanced Epithelial Immunity to a Natural Microsporidian Parasite

**DOI:** 10.1371/journal.ppat.1004583

**Published:** 2015-02-13

**Authors:** Keir M. Balla, Erik C. Andersen, Leonid Kruglyak, Emily R. Troemel

**Affiliations:** 1 Division of Biological Sciences, Section of Cell and Developmental Biology, University of California, San Diego, La Jolla, California, United States of America; 2 Department of Molecular Biosciences, Northwestern University, Evanston, Illinois, United States of America; 3 Department of Human Genetics, Department of Biological Chemistry, and Howard Hughes Medical Institute, David Geffen School of Medicine, University of California, Los Angeles, Los Angeles, California, United States of America; Stanford University, UNITED STATES

## Abstract

Microbial pathogens impose selective pressures on their hosts, and combatting these pathogens is fundamental to the propagation of a species. Innate immunity is an ancient system that provides the foundation for pathogen resistance, with epithelial cells in humans increasingly appreciated to play key roles in innate defense. Here, we show that the nematode *C. elegans* displays genetic variation in epithelial immunity against intestinal infection by its natural pathogen, *Nematocida parisii*. This pathogen belongs to the microsporidia phylum, which comprises a large phylum of over 1400 species of fungal-related parasites that can infect all animals, including humans, but are poorly understood. Strikingly, we find that a wild *C. elegans* strain from Hawaii is able to clear intracellular infection by *N. parisii*, with this ability restricted to young larval animals. Notably, infection of older larvae does not impair progeny production, while infection of younger larvae does. The early-life immunity of Hawaiian larvae enables them to produce more progeny later in life, providing a selective advantage in a laboratory setting—in the presence of parasite it is able to out-compete a susceptible strain in just a few generations. We show that enhanced immunity is dominant to susceptibility, and we use quantitative trait locus mapping to identify four genomic loci associated with resistance. Furthermore, we generate near-isogenic strains to directly demonstrate that two of these loci influence resistance. Thus, our findings show that early-life immunity of *C. elegans* against microsporidia is a complex trait that enables the host to produce more progeny later in life, likely improving its evolutionary success.

## Author Summary

Infectious diseases caused by microbes create some of the strongest forces in evolution, by killing their hosts, and impairing their ability to produce progeny. Microsporidia are very common microbes that cause disease in all animals, including roundworms, insects, fish and people. We investigated microsporidia infection in the roundworm *C. elegans*, and found that strains from diverse parts of the world have differing levels of resistance against infection. Interestingly, a *C. elegans* strain from Hawaii can clear infection but only during the earliest stage of life. This resistance appears to be evolutionarily important, because it is during this early stage of life when infection can greatly reduce the number of progeny produced by the host. Consistent with this idea, if the Hawaiian strain is infected when young, it will ultimately produce more progeny than a susceptible strain of *C. elegans*. We find that this early life resistance of Hawaiian animals is due to a combination of genetic regions, which together provide enhanced immunity against a natural pathogen, thus enabling this strain to have more offspring.

## Introduction

Infectious disease is one of the strongest drivers of evolution, generating diversification in hosts and pathogens through a dynamic co-evolutionary process of adaptation and counter-adaptation. The dynamism of these relationships is apparent in emerging infectious diseases, whereby an interaction between organisms changes from being benign to being harmful for the host [1]. Emerging diseases can have devastating effects on their hosts, and understanding how infectious diseases emerge is therefore a major goal for medicine, agriculture, and evolutionary biology.

Microsporidia are emerging pathogens that comprise a diverse phylum of more than 1400 species of fungal-related obligate intracellular parasites that are able to infect virtually all animals [2–5]. *Encephalitozoon intestinalis* is one of the many species known to infect humans, and stands out as having the smallest eukaryotic genome identified to date [6]. One consequence of the genomic reduction observed in microsporidia is their reliance on host metabolic machinery for propagation. Microsporidia commonly infect intestinal epithelial cells and can be transmitted via a fecal-oral route, although tissue tropism varies broadly. Incidences of microsporidia infection in humans were previously thought to be restricted to immunodeficient patients, but several recent studies have found an unexpectedly high prevalence among healthy people in developed countries, although the overall impact of microsporidia on the health of immunocompetent people is poorly defined [7–9]. In addition to their previously underappreciated disease-causing potential in humans, microsporidia are considered emergent pathogens of agriculturally important animals including fish and honeybees [10–12]. Despite such ubiquity, little is known about the genetic and molecular basis for pathogen defense in any host-microsporidia interaction.

Immune defense against pathogens such as microsporidia will provide evolutionary benefit if it enables hosts to produce more progeny. As such, evolutionary theory predicts that there will be less selection for immunity in post-reproductive animals [13]. The decline of immune function is termed immunosenescence, and has been observed in post-reproductive animals ranging from humans to invertebrates [14–16]. In the human population immunosenescence has been shown to be a complex trait regulated by several genetic loci [17]. Several outstanding questions remain regarding the process of immunosenescence, including its precise timing over the lifetime of an organism and how it has been shaped by pathogens over evolutionary time.

We use the nematode *Caenorhabditis elegans* as a convenient host to characterize resistance to a natural microsporidian pathogen. This pathogen is called *Nematocida parisii*, or nematode-killer from Paris, because it was isolated from wild-caught *C. elegans* from a compost pit near Paris [18]. The life cycle of *N. parisii* is similar to those of other microsporidia species, which use a specialized infection apparatus called a polar tube to invade directly into host cells, where they undergo their life cycle (S1 Fig.). In the case of *N. parisii*, spores are consumed by *C. elegans*, enter the intestinal lumen, and then invade intestinal cells. This *N. parisii* ‘sporoplasm’ becomes a meront, which replicates in direct contact with host cytosol, and then differentiates back into spores. These spores enter the host trafficking system, exit host cells via apical exocytosis back into the intestinal lumen, and return to the outside environment via defecation [19] (S1 Fig.). Wild-caught nematodes infected with *Nematocida* species have been isolated from many distinct environmental locations [18, 20], suggesting that microsporidia have imposed widespread evolutionary pressure on the defense system of *C. elegans. C. elegans* has no known professional immune cells and relies predominantly on epithelial cells as ‘non-professional’ immune cells for defense against infection [21, 22]. The *C. elegans* intestine is a relatively simple structure composed of just 20 non-renewable epithelial cells that share structural and functional similarity with human intestinal epithelial cells [23]. Thus, the natural *C. elegans-N. parisii* host-pathogen pair provides an excellent system in which to investigate epithelial defenses shaped over evolutionary time.

Here, we show that there is natural variation in *C. elegans* defense against microsporidia. We find that a *C. elegans* strain from Hawaii has enhanced resistance to *N. parisii* compared to other *C. elegans* strains. Interestingly, immunity in the Hawaiian strain occurs via clearance of intracellular infection from intestinal epithelial cells. This clearance of *N. parisii* represents an impressive example of non-professional immune cells being able to not just resist but eliminate microbial infection. Intriguingly, only very young (first larval stage L1) animals are able to clear infection; Hawaiian animals rapidly lose this ability even before they reach reproductive age. We find that *N. parisii* infection impairs progeny production only when animals are inoculated at the L1 stage, and not when they are inoculated at the later L4 stage, providing a likely evolutionary explanation for why there is enhanced resistance only in L1 animals. Enhanced resistance confers a selective advantage, allowing Hawaiian animals to outcompete a susceptible host strain in only a few generations. Finally, we determine that Hawaiian resistance to *N. parisii* is a complex multigenic trait that maps to at least four quantitative trait loci (QTL), and we show with near-isogenic lines (NILs) how two of these loci contribute to resistance. These results demonstrate that intestinal epithelial cells in a wild *C. elegans* strain can eliminate intracellular microsporidia infection. Interestingly, this complex trait acts only at a developmental stage in which it promotes progeny production, and thus likely provides an evolutionary benefit to the host.

## Results

### Natural variation in survival and resistance to microsporidia infection

**Figure 1:**
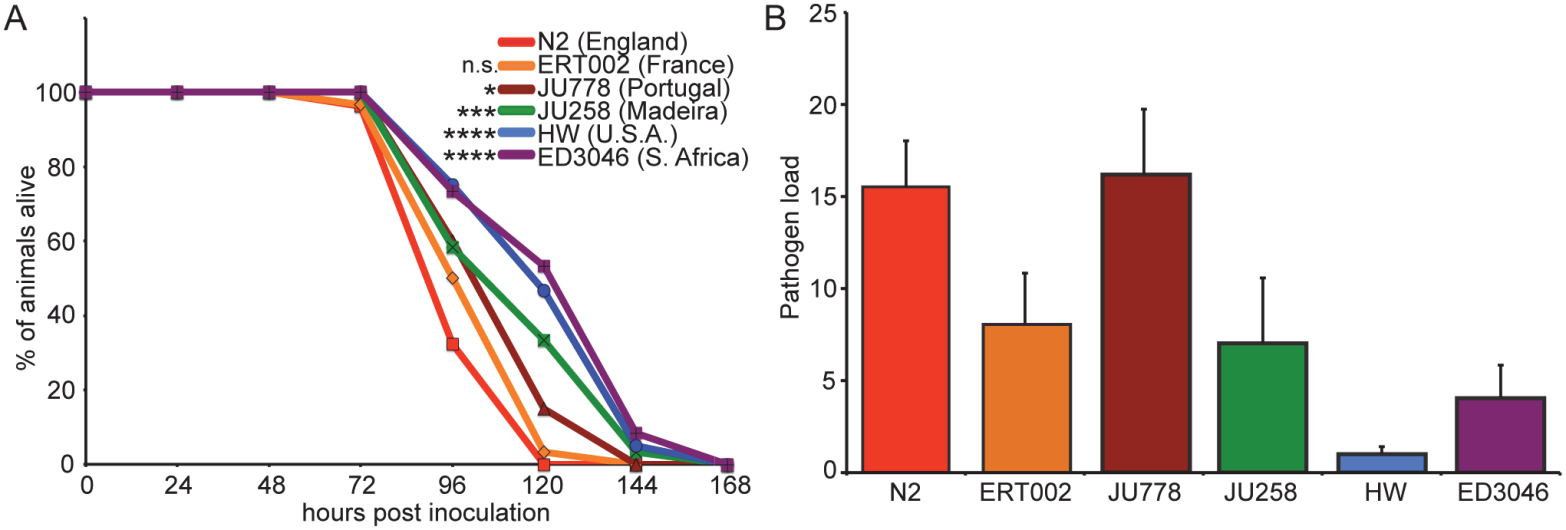
Natural variation in *C. elegans* response to *N. parisii* infection. (A) Survival curves of six *C. elegans* strains upon infection by *N. parisii*. Data are mean values of three plates containing 30 animals each and are representative of two independent experiments. Significance was measured by the Log-rank (Mantel-Cox) test comparing each strain to N2, with asterisks indicating *p*-values where *<0.05, ***<0.001, and ****<0.0001. (B) Pathogen load 30 hpi measured by qRT-PCR targeting an *N. parisii* small subunit rRNA, normalized to a *C. elegans* small subunit rRNA (see Materials and Methods for details). Data are mean values of four biological replicates from two independent experiments with error bars denoting standard deviation (SD). Differences among means are statistically significant (P<0.05) by one-way ANOVA.

To determine whether there is natural variation in the ability of *C. elegans* to defend against its natural intracellular pathogen *N. parisii*, we investigated infection in a collection of geographically diverse *C. elegans* strains. *N. parisii* has been shown to shorten the lifespan of *C. elegans* due to a lethal intestinal infection [18], and so we first examined survival upon infection using six strains that represent diverse haplotypes from a global collection of *C. elegans* [24]. We infected populations of synchronized first larval stage (L1) animals with *N. parisii* spores and quantified the percentage of animals alive over time. In these experiments, we observed variation in the survival during infection with time to 50% of animals dead (TD50) ranging from 90 to 120 hours among the various *C. elegans* strains (Fig. 1A). The standard *C. elegans* N2 laboratory strain from Bristol, England had a relatively short TD50 of about 90 hours. This strain has been passaged under laboratory conditions for decades, and interestingly, did not have significantly different longevity than the *C. elegans* wild-caught strain ERT002 from Paris, France, which has been passaged very little under laboratory conditions. Also of note, ERT002 harbored the original isolate of *N. parisii* [18], indicating that it had been exposed to pressure from microsporidia in the wild in the recent past. Strains JU778 from Portugal and JU258 from Madeira had intermediate levels of survival upon infection. By contrast, strain ED3046 from South Africa and strain CB4856 from Hawaii, USA (hereafter designated HW) survived significantly longer than the other strains. Furthermore, we observed that all strains lived longer in the absence of infection (S2 Fig.). N2, HW, and JU258 had similar lifespans in the absence of infection, which were on average slightly longer than those of ERT002, JU778, and ED3046. Thus, there is natural variation in survival of *C. elegans* upon infection by its natural intracellular pathogen, *N. parisii*.

Variation in survival upon infection could be due to variation in resistance (the ability to control pathogen load) or tolerance (the ability to cope with infection). To discriminate between these possibilities, we measured pathogen load 30 hours post-inoculation (hpi), which corresponds to the meront stage of *N. parisii* development, before spores have formed (see S1 Fig. for *N. parisii* life cycle). To measure pathogen load, we developed a quantitative PCR assay whereby levels of *N. parisii* small subunit ribosomal RNA are measured and normalized to levels of *C. elegans* small subunit ribosomal RNA as a control. Using this assay, we observed variation in pathogen load among strains (Fig. 1B) and found that most strains that survived longer had lower pathogen load (S3 Fig.). These results demonstrate that there is natural variation in *C. elegans* resistance against infection, *i.e.* the ability of *C. elegans* to control levels of *N. parisii* pathogen load.

### Young HW animals exhibit enhanced resistance to infection, which is rapidly lost during development

Given the phenotypic extremes exhibited by N2 and HW, we further investigated the variation in pathogen resistance between these two strains. In the experiments described above, we found that HW was highly resistant to a strain of *N. parisii* that was isolated in the state of Hawaii (strain ERTm5—See Materials and Methods). We next infected N2 and HW with a strain of *N. parisii* that was isolated in Paris, France (strain ERTm1) to determine whether HW *C. elegans* were also more resistant to a strain of *N. parisii* isolated from a distant geographical location. Indeed, we found that HW also lived longer and was more resistant than N2 when infected with the *N. parisii* strain from France (S4 Fig.). All subsequent experimentation was performed with the *N. parisii* strain from Hawaii.

**Figure 2:**
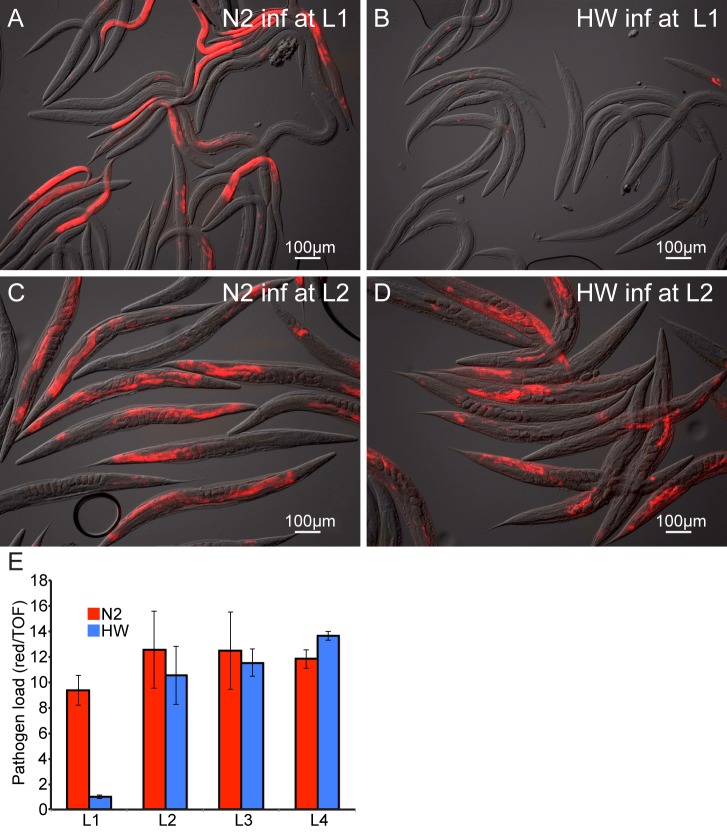
Age-dependent variation in resistance to *N. parisii* infection. Representative images of N2 (A,C) and HW (B,D) infected with *N. parisii* as L1s (A,B) or L2s (C,D) and stained 30 hpi by FISH with an *N. parisii*-specific rRNA probe (red). (E) Pathogen load in N2 and HW 30 hpi across the four larval stages. Infection is quantified with a COPAS Biosort by measuring the mean red *N. parisii* FISH signal in individual animals normalized to nematode size (assessed by time-of-flight, or TOF). Graph shows mean values across three independent experiments with error bars as SD. For each experiment, the mean was determined from two biological replicates, each containing 1500 animals.

To confirm via a different assay that HW animals are more resistant to infection than N2 animals, we examined pathogen load in N2 and HW animals using a fluorescence *in situ* hybridization (FISH) assay with a fluorescent probe that targets the *N. parisii* small subunit rRNA. Consistent with the qPCR results (Fig. 1B), we found that pathogen load 30 hpi was much lower in HW animals compared to N2 animals (Fig. 2A–B).

One potential reason for decreased pathogen load of HW animals is that they may simply feed less than N2 animals and thereby ingest a lower initial inoculum of *N. parisii* spores. To examine this possibility, we compared the feeding rate between N2 and HW at the L1 stage by inoculating animals with GFP-labeled *E. coli* together with *N. parisii* spores and measuring fluorescent accumulation in the intestinal lumens of individuals over time. These experiments revealed that HW L1 animals did not feed less than N2 L1 animals and in fact fed slightly more (S5 Fig.). Thus, lower pathogen load in HW animals is not simply caused by a lower rate of feeding and a lower initial inoculum of pathogen.

The experiments described above were performed with animals infected at the first larval stage of development, although previously we had described that N2 *C. elegans* are susceptible to infection by *N. parisii* at all four larval stages (L1 through L4), as well as the adult stage [18]. Interestingly, we found that the difference in pathogen load between N2 and HW was vastly reduced when animals were inoculated with *N. parisii* at the L2 stage, compared to animals that were inoculated at the L1 stage (Fig. 2C–D). We quantified pathogen load by FISH staining and COPAS Biosort analysis of a population of animals 30 hpi at each of the four larval stages and found that the differences between N2 and HW were restricted to infections initiated at the L1 stage (Fig. 2E). These results indicate that young HW animals are much more resistant to infection than young N2 animals, but this enhanced pathogen resistance of HW animals is rapidly lost with age.

### Young HW animals can clear infection, whereas N2 animals and older HW animals cannot clear infection

**Figure 3:**
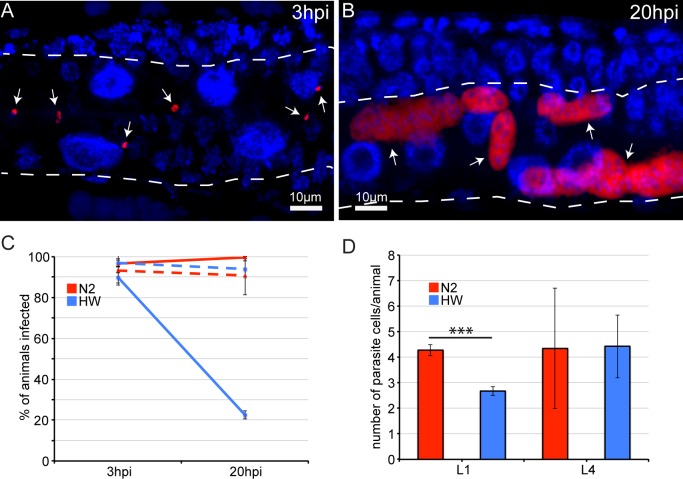
Age- and strain-dependent variation in clearance of *N. parisii* infection. (A) Early *N. parisii* sporoplasms in an N2 animal 3 hpi, fixed, and stained for DNA with DAPI (blue) and for *N. parisii* rRNA with FISH (red). (B) *N. parisii* multi-nucleate meronts in an N2 animal 20 hpi. (A, B) Arrows indicate individual parasite cells, dotted white line indicates the intestine. (C) Percentage of animals that are infected at 3 hpi and 20 hpi. Solid lines show percentages when animals are inoculated at the L1 stage, and dashed lines show percentages when animals are inoculated at the L4 stage. The mean percentage of each condition from three independent experiments is shown with error bars as SD. Each experiment had at least 100 animals per condition. (D) Mean number of parasite cells measured by FISH 3 hpi in infected L1 and L4 stage animals from three independent experiments with error bars as SD. Asterisks indicate significance (P<0.001) by t-test. Each experiment had at least 100 animals per condition.

The pathogen resistance of HW animals could be caused by an inability of *N. parisii* to invade and establish an infection in these animals, or by the ability to limit or clear an infection once it has been established. The results from our feeding experiments with fluorescent *E. coli* indicated that HW animals receive a similar initial inoculum of pathogen in their intestinal lumens (S5 Fig.), but it remained possible that the pathogen may be less able to invade and establish an infection inside intestinal cells of HW animals. To investigate this possibility, we analyzed intracellular infection at a very early stage. Previously, we had identified the earliest signs of *N. parisii* invasion and intracellular growth at 8 hpi [25] (and see life cycle in S1 Fig.), and here we show that intracellular *N. parisii* parasite cells can be identified even earlier at 3 hpi, by visualization of small, mono-nucleate *N. parisii* ‘sporoplasms’ inside *C. elegans* intestinal cells (Fig. 3A). These sporoplasms then develop into larger, multi-nucleate meronts by 20 hpi (Fig. 3B). We quantified this infection and found that approximately 90% of animals in a population of either N2 or HW animals had at least one intracellular pathogen cell in their intestines (Fig. 3C). To further quantify this initial invasion and infection, we counted the number of parasite cells per animal at 3 hpi and found that this number was slightly lower in HW animals (Fig. 3D). The fact that a similar percentage of N2 or HW animals is infected at 3 hpi lends further support to the hypothesis that the variation in resistance is not caused by differences in the rate of pathogen exposure or invasion but rather by an enhanced resistance in HW animals that acts post-invasion to mediate clearance of infection.

Next, we directly assessed whether HW *C. elegans* can clear an infection by comparing infection at different time points. Because it is necessary to fix and stain infected animals to conclusively identify pathogen cells, we cannot track a single parasite cell over time in the same animal. Instead, we analyzed animals sampled from the same infected population over time. In the previously described experiments analyzing infection at 30 hpi (Figs. 1 and 2), *C. elegans* animals were inoculated with infectious *N. parisii* spores and were continuously exposed to these spores throughout the course of the experiment. Under these conditions, all animals in a population will eventually become infected. To create conditions in which it may be possible to observe an animal clear an infection that has already been established, we developed a ‘pulsed-inoculation’ assay. Specifically, we took half of the animals from a population at 3 hpi that had been analyzed as described above and re-plated them in the absence of spores. We then harvested these animals at 20 hpi, fixed, FISH-stained to label pathogen cells and then determined the percentage of animals exhibiting infection, where 0% means no animals in the population had infection and 100% means that all animals in the population had at least one pathogen cell present. Strikingly, the percentage of HW animals that showed any evidence of infection was dramatically decreased from 90% at 3 hpi to only about 20% at 20 hpi, indicating that most animals that were infected at 3 hpi were able to clear the infection by 20 hpi (Fig. 3C). By contrast, N2 animals did not show a lower percentage of animals infected at 20 hpi, indicating they were not able to clear infection. Furthermore, when animals were inoculated at the L4 stage, neither N2 nor HW animals were able to clear the infection (Fig. 3C). Thus, it appears that young HW animals can clear an intracellular *N. parisii* infection from their intestinal epithelial cells, but they lose this ability before reaching a reproductive age.

### Resistance in young HW animals reduces mortality, maintains fecundity, and confers a selective advantage during infection

**Figure 4:**
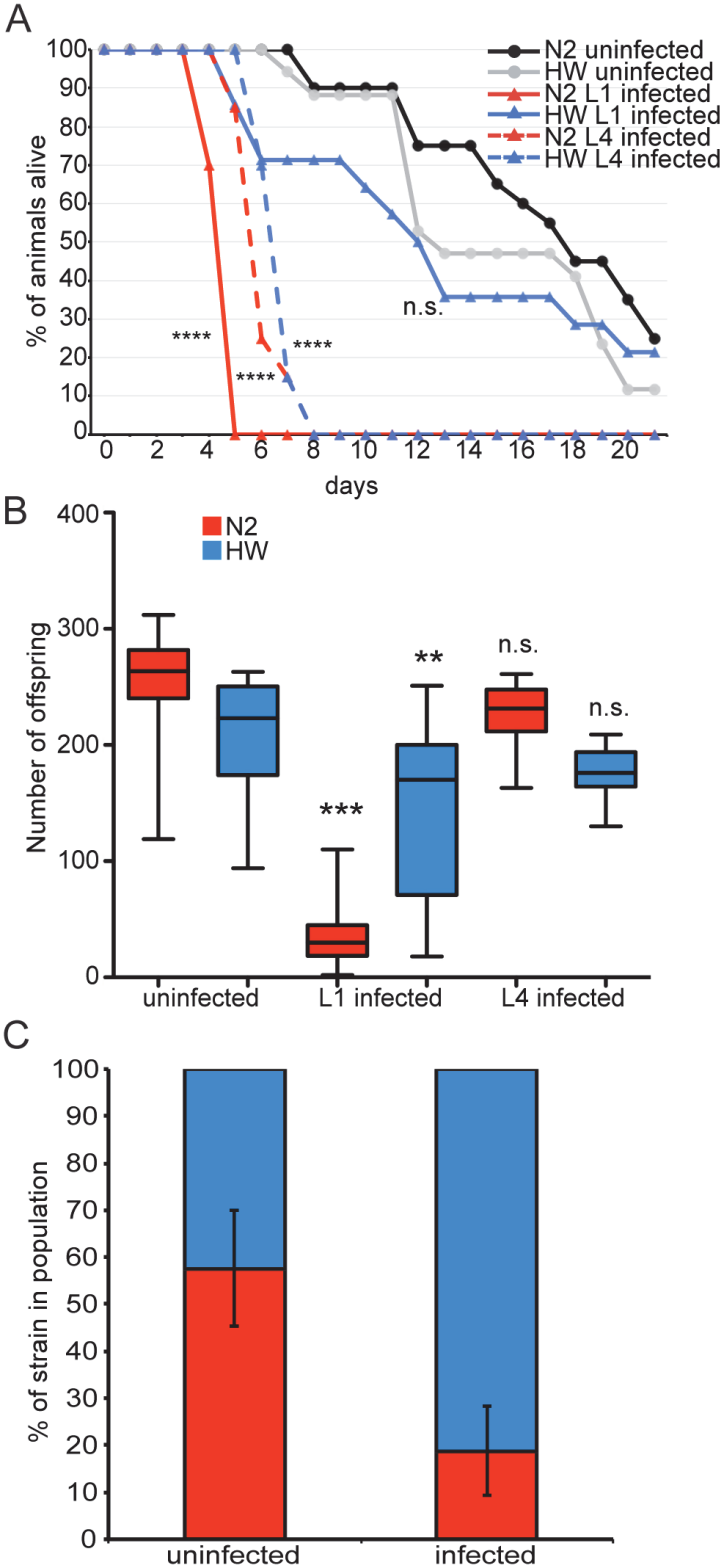
Survival, fecundity, and competition between N2 and HW strains in the presence and absence of infection. (A) Survival curves of uninfected N2 and HW animals, animals infected at the L1 stage, and animals infected at the L4 stage plotted on the number of days post L1 stage. Significance was measured by the Log-rank (Mantel-Cox) test comparing infected groups to uninfected of the same strain with asterisks indicating *p*-values where ****<0.0001. (B) Lifetime fecundity of uninfected and infected animals is shown. Conditions were compared by one-way ANOVA and Tukey’s multiple comparison test with significance reported for comparisons with uninfected controls. Asterisks indicate *p*-values, where **<0.001 and ***<0.0001. Data for (A) and (B) are from the same animals. Both (A) and (B) are a representative of two independent experiments each with 20 animals per condition. n.s. means not significant. (C) Competitive outcome of an environment shared by N2 and HW in the absence and presence of infection. The proportion of the population that is N2 is shown in red and HW in blue. Mean proportions across three independent experiments are shown, each with three biological replicates, with error bars as SD from all nine biological replicates.

One potential driver of age-specific resistance could be variation in the selective pressure that is applied by infection at different ages. Thus, we investigated the relative fitness of N2 and HW animals exposed to pathogen at different ages, focusing first on survival as a measure of fitness. In our results described above, HW animals lived about 33% longer than N2 animals during infection (Fig. 1A). In these experiments, animals were inoculated as L1 animals and then exposed to pathogen throughout their lifetimes. In order to more closely compare differential immunity to exposure at different ages, we performed the ‘pulsed-inoculation’ for three hours, removed animals from pathogen and then measured lifespan. With this ‘pulsed-inoculation’ introduced at the L1 stage, HW animals lived two and half times longer than N2 animals (Fig. 4A). Strikingly, HW animals inoculated as L1 animals had relatively little decrease in survival compared to uninfected HW animals (Fig. 4A). By contrast, N2 animals inoculated as L1 animals had dramatically decreased survival compared to uninfected N2 animals. Thus, the early life immunity of HW L1 animals was sufficient to nearly eliminate the negative impact of pathogen exposure on survival during this time. Interestingly, no significant difference in survival between N2 and HW animals was observed when pathogen inoculation occurred at the L4 stage. In this experiment, both N2 and HW animals died much more quickly than uninfected controls.

To further investigate how age-specific resistance of HW animals may affect fitness, we investigated overall progeny production, which is a key driver of evolutionary success. We found that progeny production for both the N2 and HW strains was not significantly different in animals inoculated with pathogen at the L4 stage compared to animals that were not exposed to pathogen. However, inoculation at the L1 stage led to a significant reduction in lifetime fecundity. In particular, N2 had drastically fewer progeny, while HW had only slightly fewer progeny (Fig. 4B). Thus, HW immunity at the L1 stage improves lifetime fecundity and is likely to improve evolutionary success. By contrast, resistance at the L4 stage does not appear to be important for evolutionary success, given that progeny number is not significantly reduced by pathogen inoculation at this stage.

Both N2 and HW have reduced lifetime fecundity when infected at the L1 stage, but infected HW animals have significantly more progeny than infected N2 animals. We tested to see if this difference confers a competitive advantage to HW in an environment shared with N2. L1 stage animals were inoculated with spores for three hours and then grown to the L4 stage in the absence of spores, followed by plating of equal numbers of N2 and HW animals on a shared plate. The population was then expanded to saturation and analyzed for the relative abundance of each *C. elegans* strain within the population. In uninfected populations the ratio of N2 to HW animals was 58% to 42%, respectively (Fig. 4C). In the presence of pathogen, the ratio of N2 to HW animals shifted to 19% and 81%, respectively (Fig. 4C). Taken together, these experiments demonstrate that the enhanced resistance of young HW animals confers a selective advantage over N2 animals in a laboratory setting.

### The HW phenotype of enhanced immunity is dominant to the N2 phenotype

Having established phenotypic variation in resistance to microsporidia infection, we sought to characterize the underlying genetic variation. Several phenotypic differences between N2 and HW have previously been investigated, and the causative genes responsible for those differences have been identified [26–34]. In particular, a variant in the *npr-1* gene, which encodes a neuropeptide Y-like G-protein-coupled receptor, is known to mediate several fitness-related differences between N2 and HW in a laboratory setting, including lifetime fecundity and avoidance of the human pathogen *Pseudomonas aeruginosa* [26, 34]. To determine whether *npr-1* is responsible for the differences in resistance to microsporidia, we measured pathogen load in N2 and HW strains harboring an introgressed *npr-1* locus from the other strain and found no significant differences between the introgressed strains and the parental strains (S6 Fig.). Furthermore, a deletion mutation for *npr-1* in the N2 background had similar pathogen load as the N2 strain (S6 Fig.). Thus, the *npr-1* gene does not appear to be responsible for the enhanced resistance to microsporidia infection of HW animals compared to N2 animals.

The increased resistance of HW animals to infection by *N. parisii* could be caused by the absence of a host factor important for *N. parisii* growth or by the presence of an increased host immune response. To distinguish between these two models, we examined whether the HW resistance phenotype was dominant or recessive to the N2 phenotype. We tested the F1 heterozygous progeny from a cross between N2 and HW for pathogen load by FISH and found that heterozygotes were as resistant as HW homozygotes (S7 Fig.), indicating that resistance is dominant. Together with the data on clearance of infection, these results support the model that HW has an increased immune response to *N. parisii* infection compared to N2.

### Resistance to microsporidia infection is a complex trait

**Figure 5:**
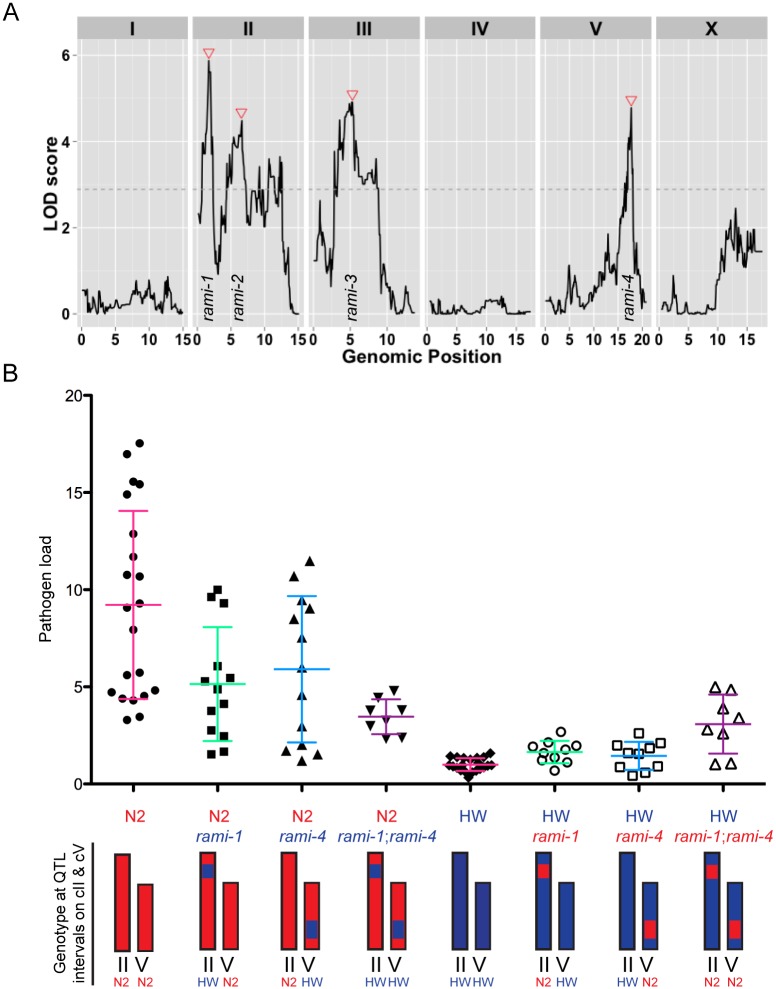
Linkage mapping results for N2 and HW resistance to *N. parisii* infection. (A) Logarithm of the odds (LOD) ratios for linkage between pathogen load and genomic position in RIAILs. Dotted line is the 5% genome-wide significance threshold obtained after 10,000 permutations of the phenotype data. Red triangles indicate *rami* loci. (B) Pathogen load measured by qRT-PCR in parental strains and near-isogenic lines (NILs) bearing the QTL intervals for chromosome II and/or chromosome V. Graphed are the mean values with error bars as SD from at least eight replicates over four experiments. Chromosomes are drawn red for N2 genotype and blue for HW genotype. The written genotype describes markers at the indicated QTL.

Next, we sought to identify the number and location of the genetic regions contributing to the variation in immunity between N2 and HW animals. We used quantitative genetic analyses to map the causal quantitative trait loci (QTL) by infecting 179 recombinant inbred advanced intercross lines (RIAILs) between the N2 and HW strains [35] and measuring pathogen load 30 hpi by qRT-PCR (S1 Table). Pathogen load values for RIAILs varied continuously and were generally well bounded by the parental values (S8 Fig.). Replicate data from the parents and all RIAILs indicated that the broad-sense heritability of resistance was 69%, signifying that much of the variation in resistance is caused by genetic factors. Single-marker regression revealed four loci on chromosomes II, III, and V that are associated with variation in resistance (Fig. 5A and S2 Table). RIAILs bearing the HW allele at these loci had significantly lower pathogen loads than those carrying the N2 allele. We named these loci Resistant Against Microsporidia Infection (*rami*): *rami-1*, *rami-2*, *rami-3*, and *rami-4*. Together, these four genetic loci account for 51% of the phenotypic variance. Thus, the *rami* QTL appear to explain the majority (51/69 = 74%) of the N2-HW genetic variance.

To confirm that the genetic loci identified by QTL analysis could influence pathogen resistance, we made and tested NILs, which bear either an interval from the N2 strain introgressed into the HW strain or an interval from the HW strain introgressed into the N2 strain. We investigated the *rami-1* and *rami-4* loci, which should account for about 15% and 12% of the phenotypic variance respectively (S2 Table). We generated NILs for *rami-1* and *rami-4* where the N2 interval was introgressed in the HW strain and vice versa. We then infected these strains with *N. parisii* and quantified pathogen load by qRT-PCR. Compared to the N2 strain, NILs in the N2 background with the *rami-1* or *rami-4* locus from HW had on average a 44% or 36% reduction in pathogen load, respectively (Fig. 5B). When both *rami-1* and *rami-4* from HW were present in the N2 background there was a 62% reduction in pathogen load compared to N2. The opposite effect was seen for NILs that were made in the HW background with *rami-1* or *rami-4* from N2; *i.e.*, these animals were more susceptible than HW. Compared to the HW strain, NILs with *rami-1* or *rami-4* from N2 were 39% and 31% more susceptible, respectively, and 67% more susceptible when *rami-1* and *rami-4* were combined (Fig. 5B). Additionally, we tested the *rami-4* NIL in the N2 background for pathogen load with the FISH assay and found that with this assay as well, the *rami-4* locus made N2 significantly more resistant to infection (S9 Fig.). Altogether, our results indicate that the *rami-1* and *rami-4* loci both additively promote *C. elegans* resistance to *N. parisii*.

## Discussion

Our findings demonstrate that there is natural variation in *C. elegans* host defense against microsporidia infection. We used variation between the N2 and HW strains to characterize the phenotypic and genetic basis of resistance to *N. parisii*. Surprisingly, we found that intestinal epithelial cells can clear intracellular infection in the HW strain but only when infection occurs at a young age. We observed that infection has a large negative impact on progeny production if it occurs at a very young age but not at a later pre-reproductive age, delineating one potential evolutionary reason for the age-specific resistance we identified. We used RIAILs generated from crosses between the susceptible N2 strain and the resistant HW strain to identify four QTL that contribute to a complex genetic basis of resistance to a natural intracellular pathogen.

Age-related decline in immune response has been widely observed among animals [15], although our findings of loss of immune function at such an early, pre-reproductive stage are unusual. Most studies of immunosenescence focus on reproductive or post-reproductive animals. For example, a master regulator of immune defense in *C. elegans* is the p38 MAPK PMK-1, which has been shown to functionally decline around day six of adulthood [16], after reproduction has ended. In addition, the *C. elegans* JNK-like MAPK KGB-1 has a reversal in protective function beginning in adulthood [36]. Here, we made the surprising observation that the enhanced resistance of the HW strain to *N. parisii* infection is limited to very young animals, and that immunity to this pathogen declines well before animals have begun adulthood and production of progeny. Our analysis suggests that the absence of enhanced immunity in older, albeit pre-reproductive, HW animals may have been shaped by weakened selective pressure. Employing a strong immune response may have negative consequences, including metabolic costs and the potential for self-damage. In the absence of selective pressure imposed by infection on progeny production that we observed in older larvae, maintaining a robust immune response may be superfluous and costly to the evolutionary success of the individual. It is surprising that infection of older pre-reproductive animals led to sharp decreases in lifespan but not to significant decreases in production of progeny. Because older animals were not able to clear infection, perhaps resources at older age are reallocated from immunity to reproduction. Our data indicate a drastic decline in immune responses to pathogens that occur earlier than those results described in other studies.

As wild *C. elegans* strains infected by microsporidia have been isolated from around the world [18, 20], it is likely that co-evolution has contributed to genetic diversity and natural variation in resistance. Researchers have isolated strains of *C. elegans* from six continents, and the genetic diversity among these strains was recently documented [24]. The N2 and HW strains are highly divergent from each other, and we found that they vary in resistance to *N. parisii*. The enhanced resistance of the HW strain may incur costs that make it less fit in the absence of infection. Our data on relative fitness support this idea, in that HW has a shorter lifespan than N2 in the absence of infection (Fig. 4A). However, this difference may be explained by variation in the *npr-1* gene [26], while the difference we see in resistance to *N. parisii* cannot (S6 Fig.). Regardless, variation between these two strains may not necessarily capture variation that is relevant to evolution in a natural setting due to adaptations that may have occurred in a laboratory setting. A case in point is the variation between N2 and HW in NPR-1-mediated behaviors, which were originally believed to be naturally derived but have since been convincingly shown to be due to a laboratory adaptation in N2 [32, 35]. As discussed above, variation in NPR-1 between N2 and HW gives N2 a fitness advantage in standard laboratory conditions. This variation confounds our ability to assess the potential costs of immunity that may be part of the resistance of the HW strain. We tested four additional wild isolates that span the geographic and genetic range of strains characterized so far and found equal proportions of relative resistance and susceptibility. The data from this limited set of strains suggest that natural variation in resistance to *N. parisii* is an ecologically relevant trait. Genetic association studies with additional strains may identify the resistance alleles that are segregating in the global population.

We found that increased survival upon *N. parisii* infection among different *C. elegans* strains generally correlated with increased pathogen resistance (ability to control *N. parisii* pathogen load). However, the *C. elegans* strain JU778 survived infection as long as the JU258 strain despite having higher pathogen load 30 hpi, suggesting that both tolerance and resistance vary among wild strains (S2 Fig.). Also supporting the variation in tolerance is the observation that the JU778 strain slightly outlived the N2 strain when infected but died faster in the absence of infection (S1 Fig.). These observations indicate that the longevity advantage of the JU778 strain may be specific to the context of *N. parisii* infection. Although there may be variation among strains in their ability to tolerate *N. parisii* infection, we focused on variation in resistance. We found that the enhanced resistance of HW is mediated by an active clearance of infection from intestinal epithelial cells. To our knowledge, clearance of intracellular pathogens by intestinal epithelial cells has not previously been demonstrated in any animal host. The cell-intrinsic immune capabilities of epithelial cells are increasingly appreciated in mammals [37]. For example, autophagy in epithelial cells can limit invasion and dissemination of bacterial pathogens [38]. Microsporidia commonly infect intestinal epithelial cells in humans. Interestingly, studies of microsporidia infection in humans suggest that intestinal infections by microsporidia might be cleared by immunocompetent people [2, 39]. It is known that the adaptive immune system is important for clearing microsporidia infections in humans, but it is attractive to speculate that human intestinal epithelial cells may also play a role in clearing infections, similar to *C. elegans* intestinal epithelial cells. Identifying the mechanisms of clearance in *C. elegans* may help elucidate the immune capacity of epithelial cells in general, which are the first line of defense against many microbial infections.

Although previous studies in *C. elegans* found that variation in resistance to the human pathogen *P. aeruginosa* was a simple trait determined predominantly by a single gene [34], we found that *C. elegans* resistance to *N. parisii* infection is a complex genetic trait. We mapped four loci that explain a large fraction of the genotypic variance and used NILs to directly confirm the effects of *rami-1* and *rami-4*. Immunity-related genes have undergone exceptional amounts of positive selection in humans and other organisms [40]. For example, genes encoding major histocompatibility locus (MHC) proteins, immune signaling proteins and antimicrobial peptides have increased in diversity over recent evolutionary time. Hundreds of genes fall within the *rami* loci, and one approach to identifying candidates for further study may be to screen for genes that display signatures of positive selection. For example, the ubiquitin-dependent proteasome adaptors encoded by F-box and MATH-BTB genes are among the most rapidly diversifying genes in the *C. elegans* genome and have unparalleled rates of birth-death evolution [41]. These genes are under strong positive selection in their substrate-binding domains but not in their Cullin-binding domains, suggesting that they have evolved to detect and degrade foreign proteins as an immune defense mechanism [41]. Ubiquitin-mediated proteolysis has been implicated in host-pathogen interactions in both plants and animals [42] and is an attractive hypothesis for how *C. elegans* might combat an intracellular invasion such as *N. parisii* infection. In support of this hypothesis, we recently found that components of the ubiquitin-proteasome system are upregulated during infection and that disrupting the ubiquitin-proteasome system or autophagy in *C. elegans* during *N. parisii* infection increases pathogen load in the N2 strain [43]. Further refinement of our infection assays, together with genetic and molecular analyses, should uncover the specific genetic polymorphisms that have evolved to produce enhanced epithelial resistance in the HW strain. Epithelial cells are critical sites of host interactions with pathogens, and we find that they can directly eliminate intracellular infection based on several genetic loci that are tailored to the success of propagating the species.

## Materials and Methods

### *C. elegans* and *N. parisii* strains

*C. elegans* strains were maintained on nematode growth media (NGM) seeded with *E. coli* OP50–1 (which is a streptomycin-resistant OP50 strain) as previously described [44]. For simplicity, this strain is referred to as OP50 throughout. To obtain starved and synchronized L1 larvae, gravid adults were bleached to isolate eggs, which then were allowed to hatch overnight at 20°C [45]. The *C. elegans* strains N2, CB4856 (HW), JU778, JU258, and ED3046 were obtained from the *Caenorhabditis* Genetics Center. ERT002 is derived from strain CPA24, which was previously isolated from a compost pile in Franconville, France and was the original strain of *C. elegans* isolated with *N. parisii* ERTm1 infection [18, 25]. Strain CPA24 was subsequently bleached to remove the infection and renamed ERT002 to conform to *C. elegans* nomenclature conventions. For mapping, we used a set of advanced intercross recombinant inbred lines generated previously with the N2 and CB4856 strains [35]. The *N. parisii* strain we used in all infection experiments except S4 Fig. was ERTm5, a *N. parisii* strain isolated from JU2055, a *Caenorhabditis briggsae* strain found in a rotting breadfruit sampled in early April 2011 by Christopher Nelson in Limahuli Gardens, Haena, Kauai (Hawaii 22.219 North, -159.5763 West). Spores were prepared and quantified as previously described [46].

### Survival assays

For survival measurements in the six *C. elegans* strains during infection (Fig. 1), synchronized L1 larvae were plated on 6 cm NGM plates seeded with OP50 and inoculated with 2 × 10^6^ *N. parisii* spores at 25°C. At 48 hpi, 30 animals were transferred to 3.5 cm plates seeded with OP50 with three plates per experiment. Live animals were quantified every 24 hours and transferred to fresh plates. For survival in N2 and HW during infection and in the absence of infection (Fig. 4), 1200 synchronized L1 larvae were inoculated with 100 μl of a 10x concentrate of an overnight OP50 culture and 2 × 10^6^ *N. parisii* spores on 6 cm NGM plates at 25°C for three hours. Animals were then washed several times to remove spores and re-plated with OP50 until 48 hpi at 20°C. Uninfected animals followed the same conditions in the absence of spores. L4-infected animals followed the same conditions but were infected for three hours at the L4 stage. 20 individuals from each condition were then plated on 3.5 cm NGM plates seeded with OP50, incubated at 20°C, and transferred to fresh plates every 24 hours until death or progeny production stopped. Mortality was recorded every 24 hours. Data were analyzed in Prism 6 with the Log-rank (Mantel-Cox) test.

### Infection assays

Animals were infected in liquid culture or on solid media. For liquid culture infections, 2000 synchronized L1 larvae in 0.5 mL of M9 buffer were plated per well in a 24-well plate. 0.5 mL of M9 buffer containing OP50 and 2 × 10^6^ *N. parisii* spores was then added to each well. Plates were incubated on a rocker at 25°C. For solid media infections, 1200 synchronized L1 larvae were inoculated with 100 μl of a 10x concentrate of an overnight OP50 culture and 2 × 10^6^ *N. parisii* spores on 6 cm NGM plates. When plating, media was evenly distributed across the entire plate. Plates were then incubated at 25°C. For infections initiated at stages other than the L1 stage, animals were plated for 24 hours at 20°C before adding spores for the L2 stage, plated for 24 hours at 25°C before adding spores for the L3 stage or plated for 24 hours at 20°C followed by 24 hours at 15°C before adding spores for the L4 stage. Samples were fixed different times post-inoculation with Tri-Reagent (Molecular Research Center) to extract RNA or with acetone to stain by FISH.

### Measurements of pathogen load by qRT-PCR

RNA was isolated by extraction with Tri-Reagent and bromochloropropane (BCP) (Molecular Research Center). 250 ng of RNA from each sample was used to synthesize cDNA with the RETROscript kit (Ambion). cDNA was quantified with iQ SYBR Green Supermix (Bio-Rad) on a CFx Connect Real-time PCR Detection System (Bio-Rad). We measured pathogen load by measuring the relative abundance of an *N. parisii* rDNA transcript normalized to a *C. elegans* rDNA transcript with the following primer sets: Np_rDNAF1: aaaaggcaccaggttgattc, Np_rDNAR1: agctctctgacgcttccttc, Ce18S_F1: ttgcgtacggctcattagag, Ce18S_R1: agctccagtatttccgcagt. Primer efficiencies were measured, and fold difference was calculated using the Livak comparative Ct method (2^-ΔΔCt^).

### Measurements of pathogen load by FISH

We used the MicroB probe conjugated to a red Cal Fluor 610 dye (Biosearch Technologies) to stain infected animals for an *N. parsii* ribosomal RNA small subunit sequence as previously described [18]. Pathogen load was measured with the COPAS Biosort (Union Biometric) or by microscopy. For analysis with the COPAS Biosort, greater than 600 animals per condition were measured for time of flight (TOF, a measure of size) and red fluorescence. Pathogen load per individual was determined by normalizing the red signal to TOF. For microscopy, samples were mounted on agarose pads with VECTASHIELD mounting medium containing DAPI (Vector Labs) and imaged using fluorescent microscopy on a Zeiss AxioImager M1 upright microscope with a 10x or 100x oil immersion objective equipped with an AxioCam digital camera and AxioVision software. Sporoplasms at 3 hpi were imaged by confocal microscopy acquired on a Zeiss LSM700 at 630x magnification using ZEN2010 software.

### Pathogen clearance assay

For L1 experiments, 1200 synchronized L1 larvae were inoculated with 100 μl of a 10x concentrate of an overnight OP50 culture and 2 × 10^6^ *N. parisii* spores on 6 cm NGM plates at 25°C for three hours. Animals were then washed several times to remove spores and half were fixed in acetone while the other half was re-plated with OP50 and incubated at 25°C until fixing 20 hpi. L4 experiments followed the same procedure, but infections were initiated at the L4 stage. Sample were stained by FISH and analyzed by microscopy. For the 3 hpi samples, 100 animals per condition were imaged at 1000x to count the number of infected individuals and the number of parasite cells per animal. For the 20 hpi samples, 100 animals were imaged at 100x to count the number of infected individuals.

### Pulsed infection and lifetime fecundity assay

1200 synchronized L1 larvae were inoculated with 100 μl of a 10x concentrate of an overnight OP50 culture and 2 × 10^6^ *N. parisii* spores on 6 cm NGM plates at 25°C for three hours. Animals were then washed several times to remove spores and re-plated with OP50 until 48 hpi at 20°C. Uninfected animals followed the same conditions in the absence of spores. L4-infected animals followed the same conditions but were infected for three hours at the L4 stage. Twenty individuals from each condition were then plated on 3.5 cm NGM plates seeded with OP50, incubated at 20°C, and transferred to fresh plates every 24 hours until death or progeny production stopped. Progeny per animal were counted every 24 hours following the L4 stage. After transferring to fresh plates, the source plates were incubated at 20°C for 24 hours to allow all eggs to hatch, then incubated at 15°C for 24 hours before counting. Data were analyzed in Prism 6 by one-way ANOVA and Tukey’s multiple comparison test.

### Competition assay

Infection was initiated as in the lifetime fecundity experiments. Once animals had reached the L4 stage, 15 N2 and 15 HW animals were added to 15 cm NGM plates seeded with 3 mL of a 10x concentrate of an overnight OP50 culture and incubated at 20°C. Animals were harvested once food was nearly depleted, which was approximately five days post-plating for uninfected populations and approximately seven days post-plating for infected populations. Each condition was repeated in triplicate per experiment over three total experiments. Genomic DNA was obtained by phenol-chloroform extraction. To determine the ratio of N2 to HW genomic DNA in the samples, we used qPCR to measure the relative abundance of a transcript in the *zeel-1* locus (deleted in HW) normalized to a *snb-1* transcript (present in both) with the following primer sets: zeel1_N2F1: gggcaattttcaaaagcaga, zeel1_N2R1: gttggtgtgctgaattttct, snb-F1: ccggataagaccatcttgacg, snb-R1: gacgacttcatcaacctgagc. Standard curves of measuring N2 and HW genomic DNA independently and at different known combined concentrations over several biological and technical replicates revealed that on average the observed ratio was 5% off from the expected ratio.

### Feeding rate measurements

For each condition, 2000 synchronized L1 larvae in 0.5 mL of M9 buffer were plated per well in a 24-well plate. 0.5 mL of M9 buffer containing unlabeled OP50 or GFP-labeled OP50 and 2 × 10^6^ *N. parisii* spores was then added to each well. Plates were incubated on a rocker at 25°C. Animals were collected each hour for three hours post-plating and mounted on agarose pads for imaging using fluorescent microscopy at a constant exposure time on a Zeiss AxioImager M1 upright microscope with a 40x oil immersion objective equipped with an AxioCam digital camera and AxioVision software. The relative amount of GFP-labeled bacteria in the intestinal lumens of animals was quantified by outlining individual animals and calculating the mean fluorescent intensity with AxioVision software.

### Pathogen load in F1 heterozygotes and homozygotes from crosses between N2 and HW

For analyzing infection in F_1_ progeny of N2 and HW crosses, 50 L4 stage males were set up with 30 L4 stage hermaphrodites overnight. Gravid hermaphrodites were bleached on 3.5 cm plates to yield eggs that hatched in the absence of food to obtain synchronized F1 progeny. OP50 was added, and synchronized larvae were inoculated with 7 × 10^5^ *N. parisii* spores and incubated at 25°C for 16 hours. Animals were fixed and stained by FISH, mounted on agarose pads, and imaged on a Zeiss AxioImager M1 upright microscope with a 10x objective equipped with an AxioCam digital camera and AxioVision software. 30 animals per condition were outlined and measured for mean fluorescent intensity in the red channel.

### QTL mapping

179 recombinant inbred advanced intercross lines (RIAILs) from a cross between the Bristol (N2) and Hawaii (CB4856) strain were phenotyped by isolating RNA from infected animals 30 hpi and measuring pathogen load by qRT-PCR (see above). N2 and HW were phenotyped in parallel for each experiment, and pathogen load in the RIAILs was normalized to N2. 21 RIAILs were phenotyped on solid media and 158 RIAILs were phenotyped in liquid media (see above for setup, data shown in S1 Table). The normalized fraction of *N. parisii* DNA of each RIAIL and the respective genotype data [35] were entered into the R statistical programming environment and processed using the qtl package [47]. The phenotypic distribution of the RIAILs had a long right tail, so QTL were mapped using non-parametric marker regression. The 5% genome-wide significance threshold was calculated based on 10,000 permutations of the phenotype data [48]. The most significant marker was used as a covariate to identify additional QTL until no more significant QTL were detected. The total phenotypic variance explained was calculated by squaring the rank-sum correlation of genotype and phenotype for each QTL. Broad-sense heritability was calculated as the fraction of phenotypic variance explained by strain from fit of a linear mixed-model of repeat phenotypic measures of the parents and some recombinant strains [49]. The total variance explained by each QTL was divided by the broad-sense heritability to determine how much of the heritability is explained by each QTL. Confidence intervals were defined as the regions contained within a 1.5 LOD drop from the maximum LOD score.

### Near-isogenic line construction

RIAILs were selected that contained N2 or CB4856 genomic regions spanning the QTL intervals for chromosome II or chromosome V. We backcrossed these regions to the appropriate parental strain at least 12 times for each line, genotyping at SNPs bounding the interval at each cross. To generate strain ERT246 *jyIR1[CB4856 > N2] II*, Qx228 males were crossed to N2 hermaphrodites and the F2’s that segregated CB4856 markers at SNPs corresponding to the physical locations 1,373,016 and 2,090,144 were selected and homozygosed. Male progeny homozygous for CB4856 markers were crossed to N2 hermaphrodites, which was repeated until the F12 generation. NILs were then genotyped at markers across the arms and centers of all autosomes to confirm that they were N2 outside of the interval. The same basic strategy was followed for generating the other three single NILs, with the chromosome V interval genotyped at physical locations 16,734,456 and 17,917,291: the Qx88 strain was used to generate strain ERT247 *jyIR2[N2 > CB4856] II*, the Qx217 strain was used to generate strain ERT248 *jyIR3[CB4856 > N2] V*, and the Qx239 strain was used to generate strain ERT249 *jyIR4[N2 > CB4856] V*. Double NILs bearing both QTL intervals from one parent in the reciprocal background were generated by crossing single NILs and genotyping at the bounding markers listed above for homozygotes in the F2 progeny. Double NIL strains are ERT250 and ERT251.

## Supporting Information

S1 Table Pathogen load data for RIAILs and parental strains used for QTL mapping. Raw pathogen load data used for mapping, organized by strain and experimental date.
(xLSx)

S2 Table Summary of the locations, sizes and phenotypic variance of QTL associated with resistance. Information on the names, chromosomal positions, and percentages of the phenotypic variance explained by the four QTL associated with variation in resistance.
(DOCx)

S1 Fig Lifecycle of *N. parisii* in *C. elegans*. Pathogen is outlined in red, nuclei of host and pathogen are shown in blue. After *C. elegans* ingests an *N. parisii* spore, the mono-nucleate contents of that spore invade an intestinal epithelial cell. Over time, a replicative multi-nucleate form called a meront is established before differentiation into new spores. These spores exit the host cell via exocytosis and transmit infection to other animals after being defecated into the environment. The approximate timing of these events are shown from infections that occur at 25°C.
(TIF)

S2 Fig Longevity of wild strains in the absence of infection. Lifespan curves of six *C. elegans* strains in the absence of infection. Data are mean values of three independent experiments, each with three replicate plates containing 30 animals for each strain.
(TIF)

S3 Fig Correlation between pathogen load and survival upon *N. parisii* infection. Data from Fig. 1A and 1B were plotted against each other, and the correlation coefficient was determined by simple linear regression analysis.
(TIF)

S4 Fig Variation in the response of N2 and HW *C. elegans* strains to infection by *N. parisii* strain ERTm1 from France. (A) Survival curves of the N2 and HW *C. elegans* strains upon infection by an *N. parisii* strain from France. Data are mean values of three plates containing 30 animals each and are representative of eight independent experiments. (B) Pathogen load at 30 hpi measured by qPCR targeting an *N. parisii* small subunit rDNA, normalized to a *C. elegans* small subunit rDNA. Data are mean values of two biological replicates from a representative of two independent experiments with error bars showing standard deviation (SD).
(TIF)

S5 Fig Measurement of feeding rate in N2 and HW L1 animals. Starved L1 animals were incubated with GFP-labeled *E. coli* (DH5α::GFP) or an unlabeled control *E. coli* strain (OP50) to measure autofluorescence. Feeding was assessed by measuring fluorescence in the intestinal lumens of individuals at 1 hpi, 2 hpi and 3 hpi. Individual values are plotted with mean and error bars as SD for each treatment.
(TIF)

S6 Fig Analysis of resistance against *N. parisii* in *C. elegans* strains that vary in the *npr-1* gene. Strains in the N2 background carrying a deletion in *npr-1(ky13)* (Cx4148) or the *npr-1* locus introgressed from HW (QG1) and a strain in the HW background carrying the *npr-1* locus introgressed from N2 (Cx11400) were infected and analyzed for pathogen load 30 hpi by qRT-PCR. Mean values are shown from biological duplicates with error bars as SD.
(TIF)

S7 Fig HW resistance to *N. parisii* is dominant to N2. Pathogen load 16 hpi measured by FISH in homozygous and heterozygous progeny of N2 and HW parents. The mean is indicated by the wide horizontal bar with SEM. Data are from three representative experiments. The male parental strain is indicated with a symbol, and the hermaphrodite parental strain has no symbol.
(TIF)

S8 Fig Pathogen load distribution in parents and RIAILs. Replicate data from parents and RIAILs across all experiments. Tukey boxplots show interquartile range (IQR) from 25th to 75th percentile with horizontal lines indicating medians. The range bars encompass all data within 1.5 IQR above and below the upper and lower IQRs, respectively. The y-axis is the pathogen load value (*N. parisii* rRNA normalized to *C. elegans* rRNA) for each replicate.
(TIF)

S9 Fig Pathogen load in NILs measured by FISH. Pathogen load measured 30 hpi by FISH in parental strains and a near-isogenic line bearing the QTL interval for chromosome V (*rami-4*). Graphed are the mean values from eight replicates over two experiments. Significance between the N2 and *rami-4* strain in the N2 background was determined by t-test. Chromosomes are drawn red for N2 genotype and blue for HW genotype. The written genotype describes markers at the indicated QTL.
(TIF)
