# Supplementary material for: A Wild C. Elegans Strain Has Enhanced Epithelial Immunity to a Natural Microsporidian Parasite
Source: PLoS Pathog. 2015 Feb 13;11(2):e1004583. doi: 10.1371/journal.ppat.1004583 (PMC4334554; doi:10.1371/journal.ppat.1004583)
Supplement: S2 Table — Information on the names, chromosomal positions, and percentages of the phenotypic variance explained by the four QTL associated with variation in resistance. (DOCx) [file ppat.1004583.s002.docx]

| QTL | Chromosome | Peak | Left boundary of confidence interval | Right boundary of confidence interval | %VE |
| --- | --- | --- | --- | --- | --- |
| *rami-1* | II | 1708761 | 1407385 | 6672335 | 15 |
| *rami-2* | II | 6538824 | 4317263 | 12008087 | 11 |
| *rami-3* | III | 5290862 | 2812977 | 8626415 | 13 |
| *rami-4* | V | 17704376 | 16837169 | 18120342 | 12 |
