# Supplementary figures and images for: A Wild C. Elegans Strain Has Enhanced Epithelial Immunity to a Natural Microsporidian Parasite
Source: PLoS Pathog. 2015 Feb 13;11(2):e1004583. doi: 10.1371/journal.ppat.1004583 (PMC4334554; doi:10.1371/journal.ppat.1004583)

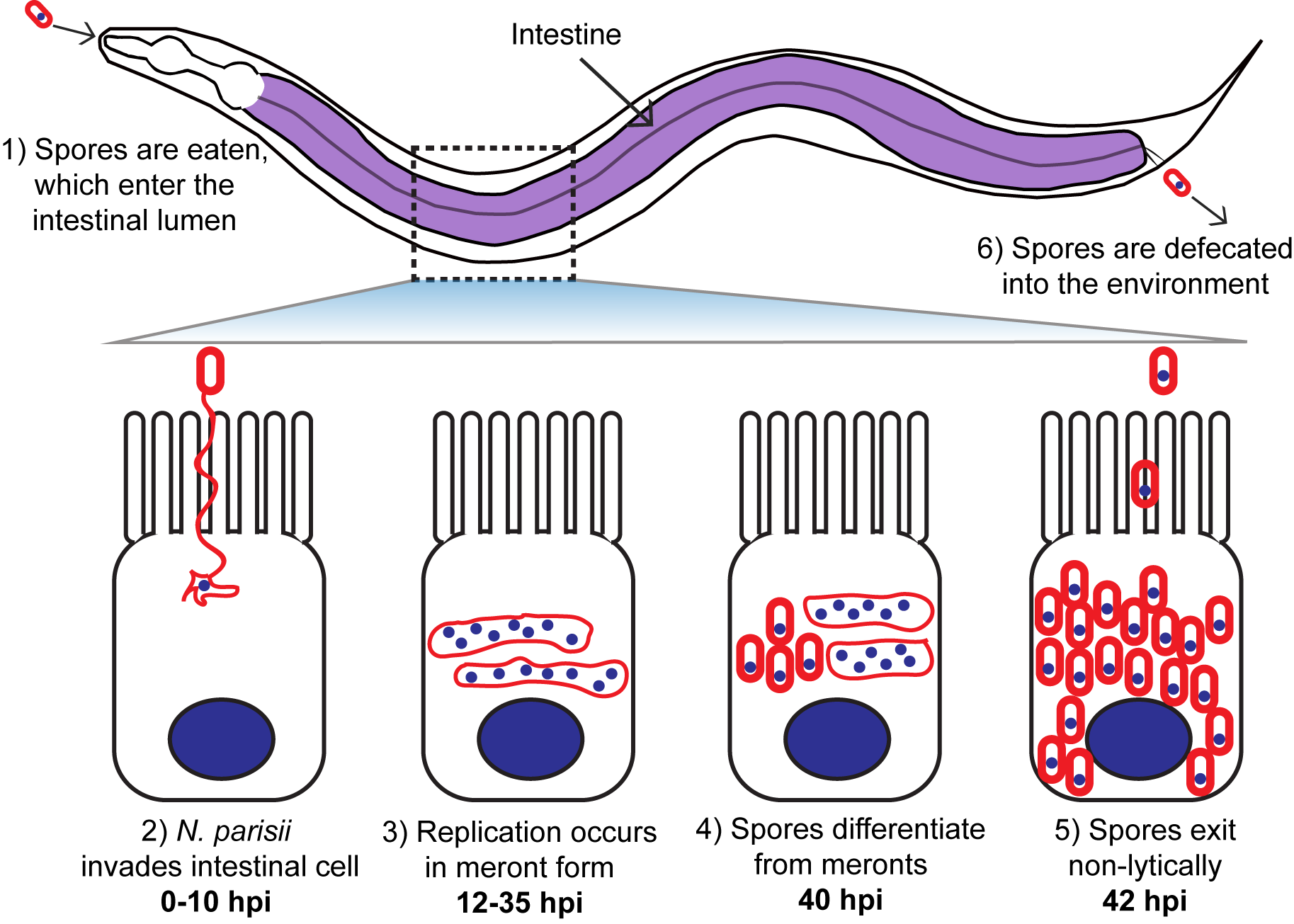

Supplement: S1 Fig — Pathogen is outlined in red, nuclei of host and pathogen are shown in blue. After C. elegans ingests an N. parisii spore, the mono-nucleate contents of that spore invade an intestinal epithelial cell. Over time, a replicative multi-nucleate form called a meront is established before differentiation into new spores. These spores exit the host cell via exocytosis and transmit infection to other animals after being defecated into the environment. The approximate timing of these events are shown from infections that occur at 25°C. (TIF) [file ppat.1004583.s003.tif]

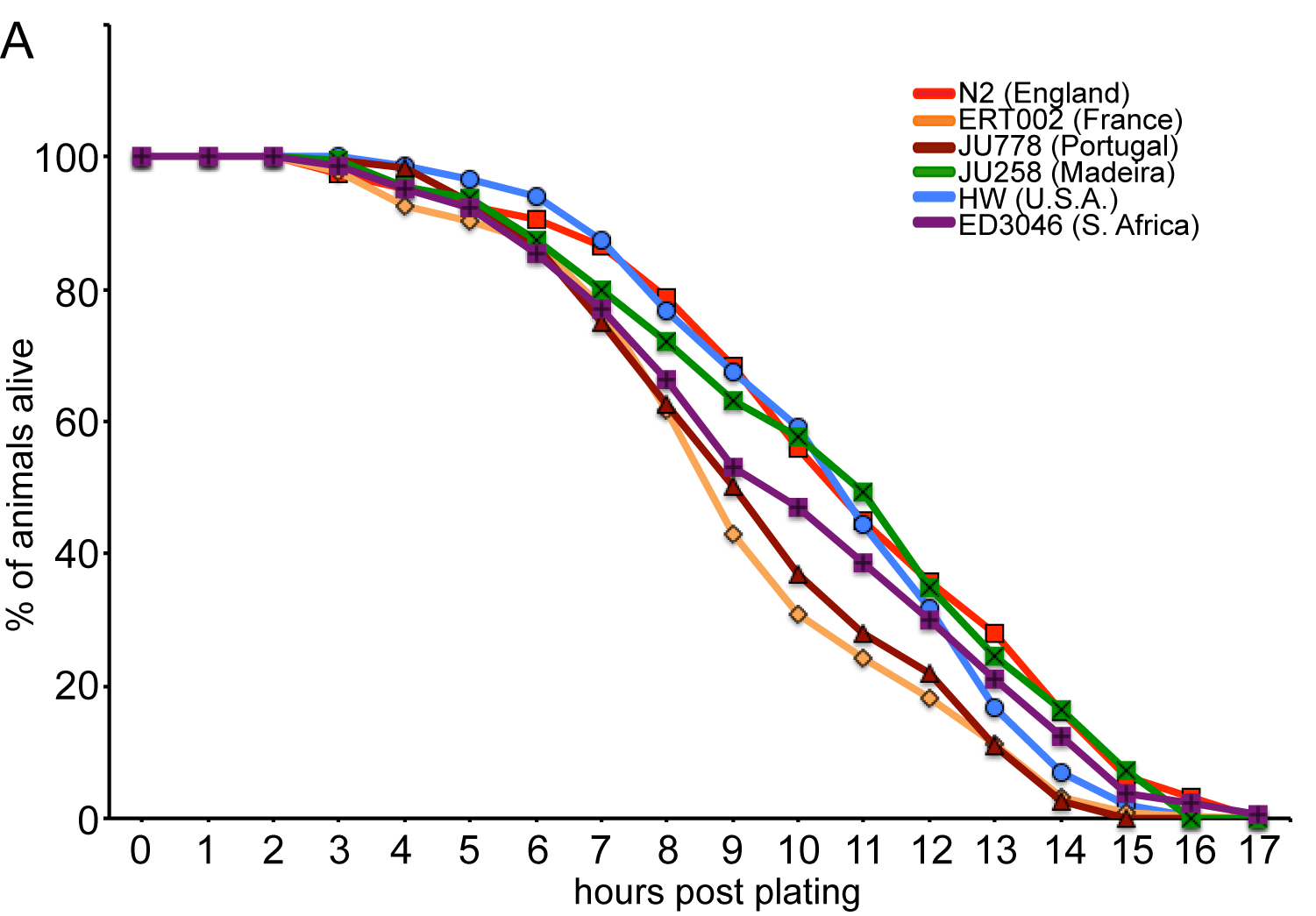

Supplement: S2 Fig — Lifespan curves of six C. elegans strains in the absence of infection. Data are mean values of three independent experiments, each with three replicate plates containing 30 animals for each strain. (TIF) [file ppat.1004583.s004.tif]

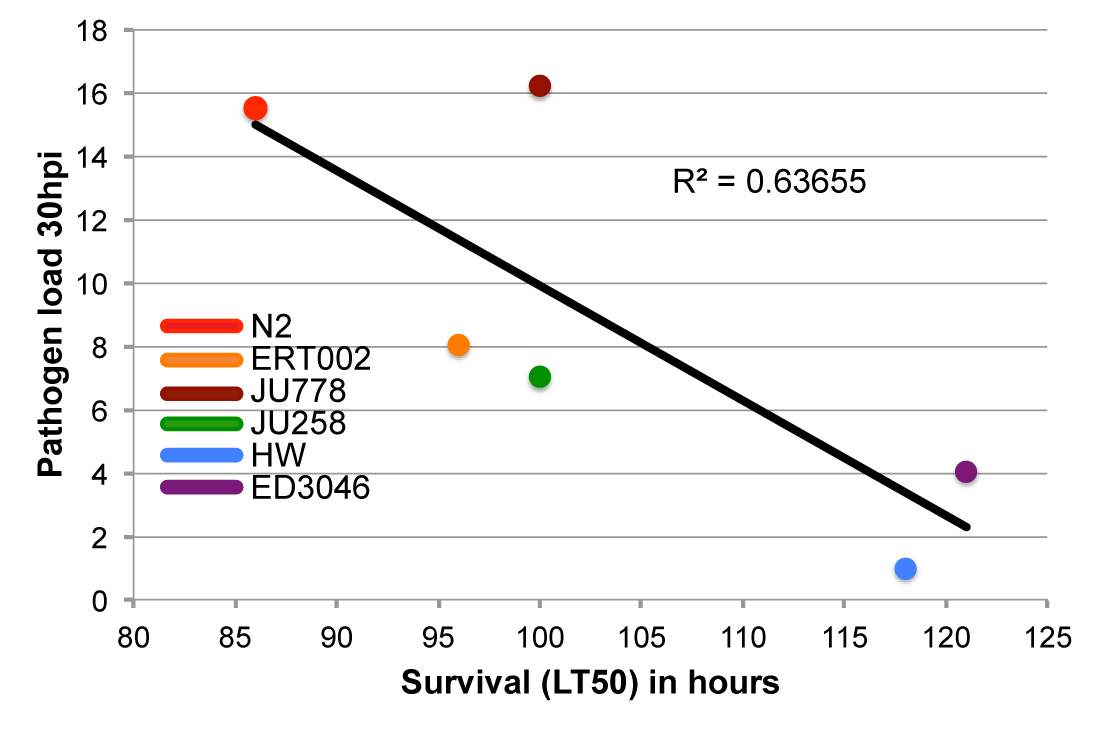

Supplement: S3 Fig — Data from Fig. 1A and 1B were plotted against each other, and the correlation coefficient was determined by simple linear regression analysis. (TIF) [file ppat.1004583.s005.tif]

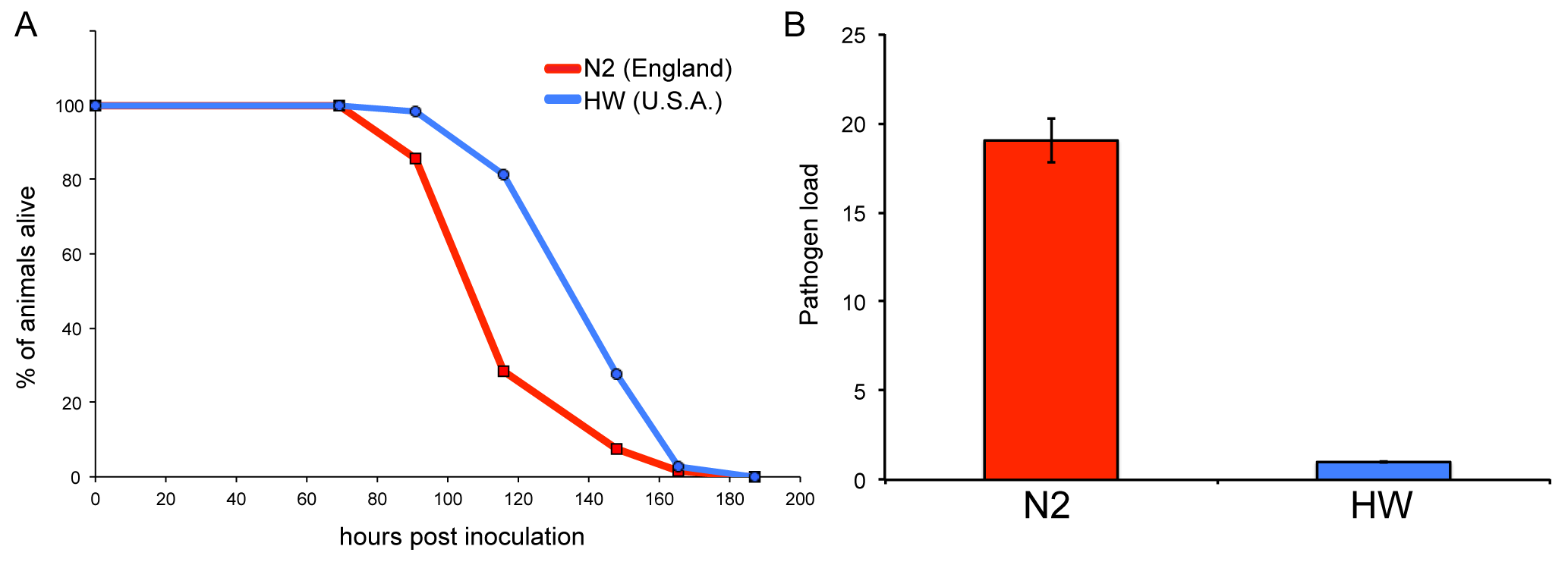

Supplement: S4 Fig — (A) Survival curves of the N2 and HW C. elegans strains upon infection by an N. parisii strain from France. Data are mean values of three plates containing 30 animals each and are representative of eight independent experiments. (B) Pathogen load at 30 hpi measured by qPCR targeting an N. parisii small subunit rDNA, normalized to a C. elegans small subunit rDNA. Data are mean values of two biological replicates from a representative of two independent experiments with error bars showing standard deviation (SD). (TIF) [file ppat.1004583.s006.tif]

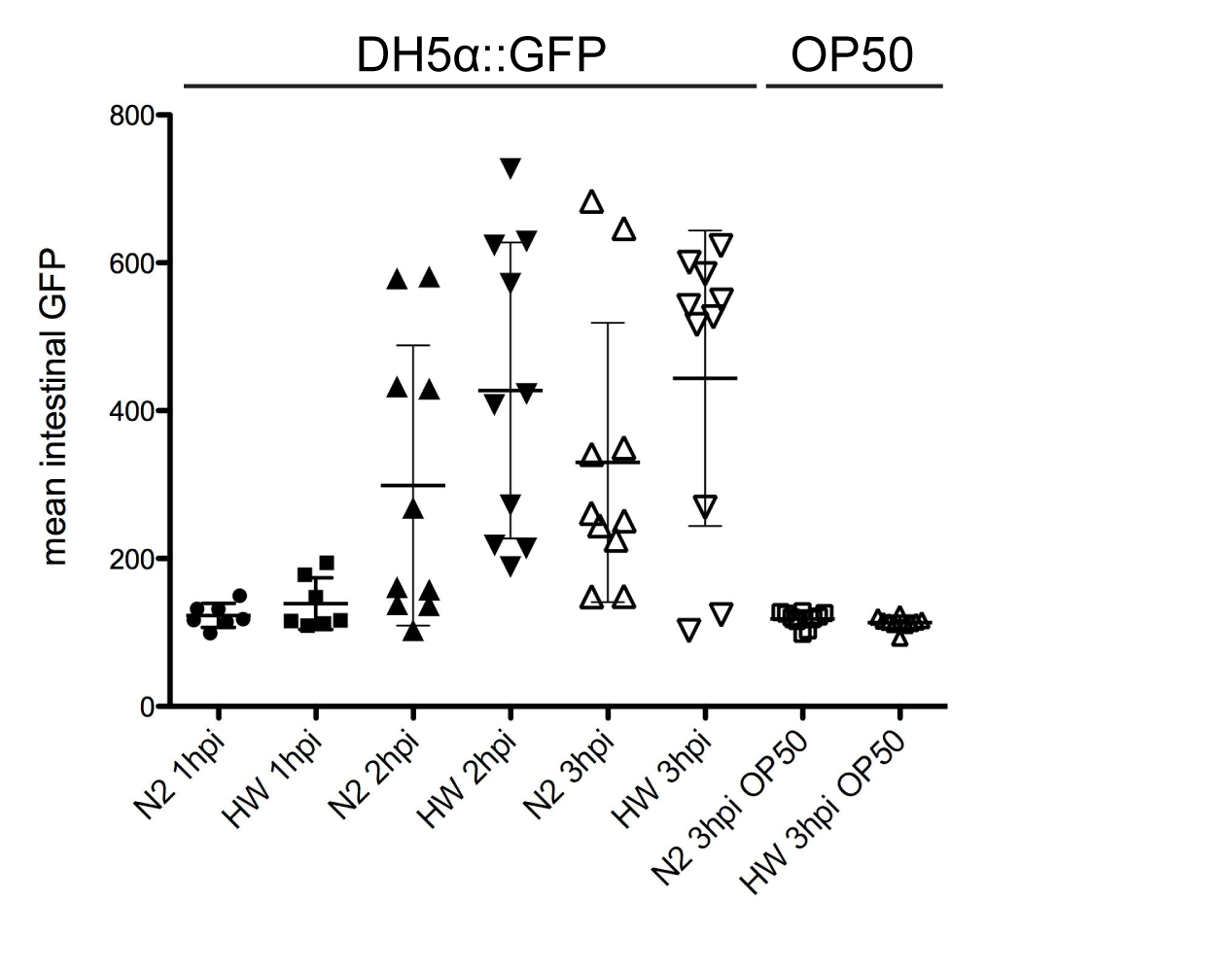

Supplement: S5 Fig — Starved L1 animals were incubated with GFP-labeled E. coli (DH5α::GFP) or an unlabeled control E. coli strain (OP50) to measure autofluorescence. Feeding was assessed by measuring fluorescence in the intestinal lumens of individuals at 1 hpi, 2 hpi and 3 hpi. Individual values are plotted with mean and error bars as SD for each treatment. (TIF) [file ppat.1004583.s007.tif]

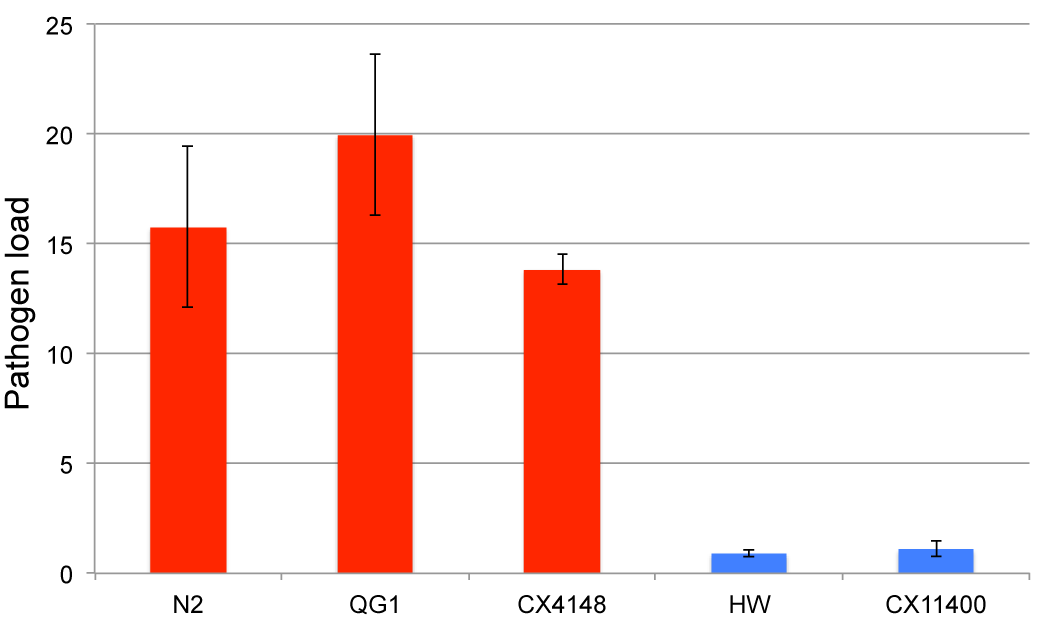

Supplement: S6 Fig — Strains in the N2 background carrying a deletion in npr-1(ky13) (Cx4148) or the npr-1 locus introgressed from HW (QG1) and a strain in the HW background carrying the npr-1 locus introgressed from N2 (Cx11400) were infected and analyzed for pathogen load 30 hpi by qRT-PCR. Mean values are shown from biological duplicates with error bars as SD. (TIF) [file ppat.1004583.s008.tif]

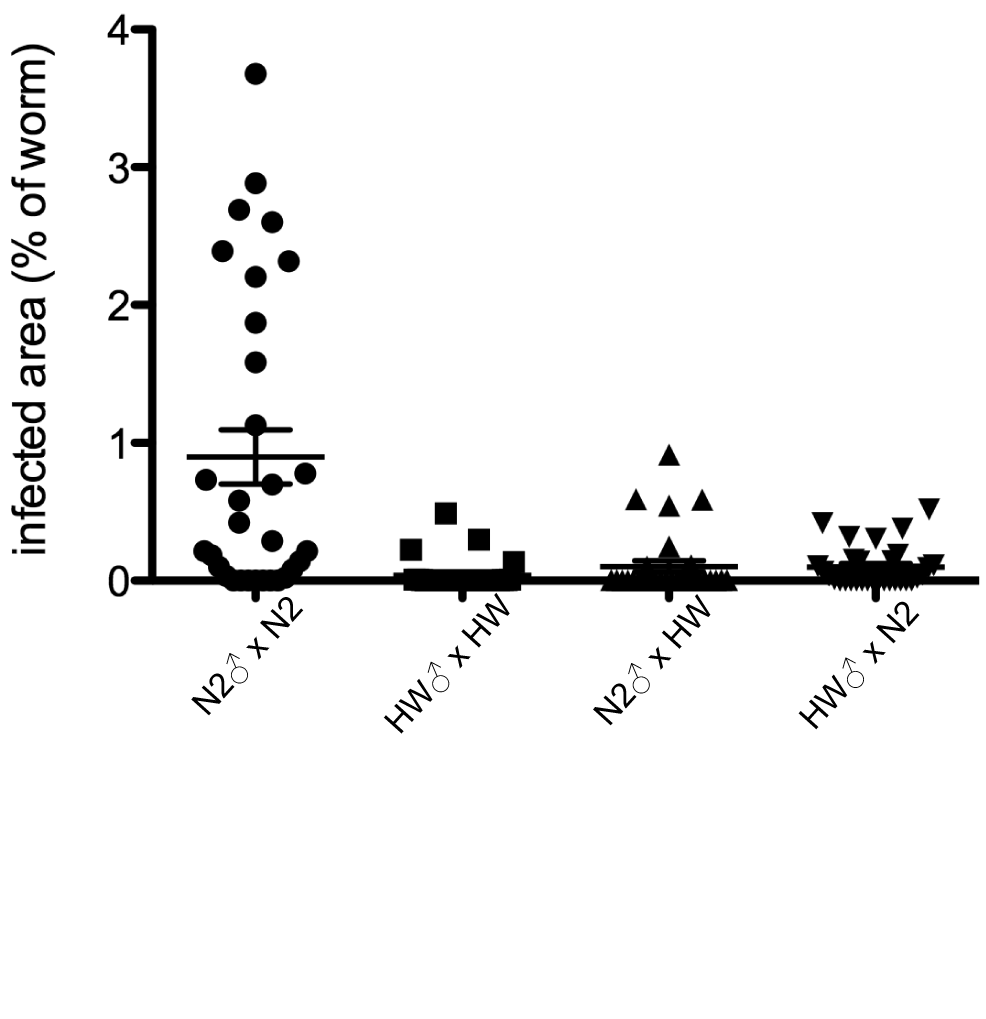

Supplement: S7 Fig — Pathogen load 16 hpi measured by FISH in homozygous and heterozygous progeny of N2 and HW parents. The mean is indicated by the wide horizontal bar with SEM. Data are from three representative experiments. The male parental strain is indicated with a symbol, and the hermaphrodite parental strain has no symbol. (TIF) [file ppat.1004583.s009.tif]

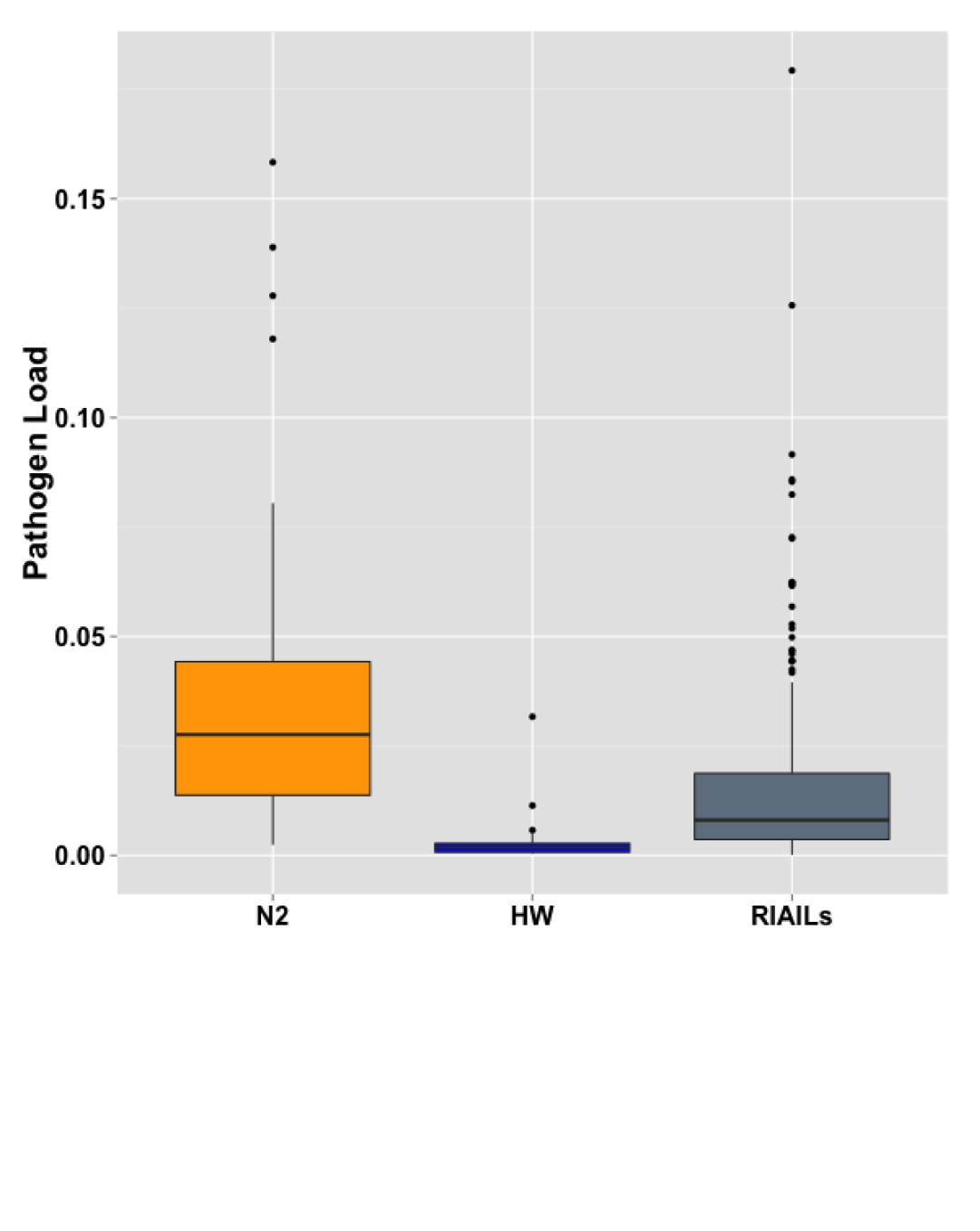

Supplement: S8 Fig — Replicate data from parents and RIAILs across all experiments. Tukey boxplots show interquartile range (IQR) from 25th to 75th percentile with horizontal lines indicating medians. The range bars encompass all data within 1.5 IQR above and below the upper and lower IQRs, respectively. The y-axis is the pathogen load value (N. parisii rRNA normalized to C. elegans rRNA) for each replicate. (TIF) [file ppat.1004583.s010.tif]

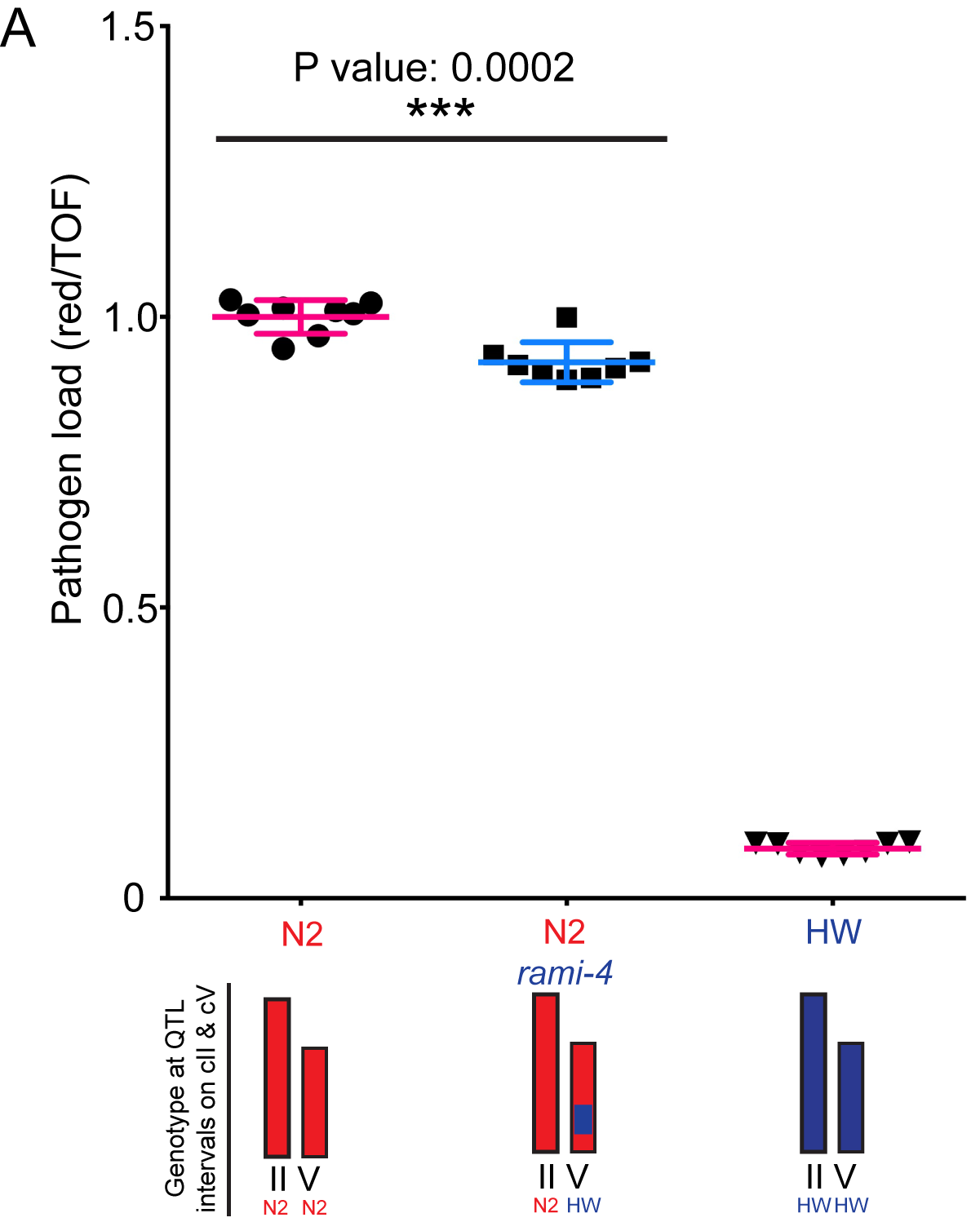

Supplement: S9 Fig — Pathogen load measured 30 hpi by FISH in parental strains and a near-isogenic line bearing the QTL interval for chromosome V (rami-4). Graphed are the mean values from eight replicates over two experiments. Significance between the N2 and rami-4 strain in the N2 background was determined by t-test. Chromosomes are drawn red for N2 genotype and blue for HW genotype. The written genotype describes markers at the indicated QTL. (TIF) [file ppat.1004583.s011.tif]
